# Targeting Neuroinflammation in Central Nervous System Diseases by Oral Delivery of Lipid Nanoparticles

**DOI:** 10.3390/pharmaceutics17030388

**Published:** 2025-03-18

**Authors:** Yuan Zou, Jing Zhang, Longmin Chen, Qianqian Xu, Sheng Yao, Hong Chen

**Affiliations:** 1 Department of Rehabilitation, Tongji Hospital, Tongji Medical College, Huazhong University of Science and Technology, Wuhan 430074, China; zouyuan@tjh.tjmu.edu.cn (Y.Z.); shengyao@tjh.tjmu.edu.cn (S.Y.); 2 Hubei Key Laboratory of Neural Injury and Functional Reconstruction, Huazhong University of Science and Technology, Wuhan 430074, China; 3 Department of Respiratory and Critical Care Medicine, The Center for Biomedical Research, NHC Key Laboratory for Respiratory Diseases, Tongji Hospital, Tongji Medical College, Huazhong University of Sciences and Technology, Wuhan 430074, China; jingzhang@tjh.tjmu.edu.cn (J.Z.); xuqianqian09@163.com (Q.X.); 4 Department of Rheumatology and Immunology, The Central Hospital of Wuhan, Tongji Medical College, Huazhong University of Science and Technology, Wuhan 430074, China; chenlongmin@zxhospital.com

**Keywords:** lipid nanoparticles, oral delivery system, neuroinflammation, blood–brain barrier, central nervous system diseases

## Abstract

Neuroinflammation within the central nervous system (CNS) is a primary characteristic of CNS diseases, such as Parkinson’s disease, Alzheimer’s disease (AD), amyotrophic lateral sclerosis, and mental disorders. The excessive activation of immune cells results in the massive release of pro-inflammatory cytokines, which subsequently induce neuronal death and accelerate the progression of neurodegeneration. Therefore, mitigating excessive neuroinflammation has emerged as a promising strategy for the treatment of CNS diseases. Despite advancements in drug discovery and the development of novel therapeutics, the effective delivery of these agents to the CNS remains a serious challenge due to the restrictive nature of the blood–brain barrier (BBB). This underscores the need to develop a novel drug delivery system. Recent studies have identified oral lipid nanoparticles (LNPs) as a promising approach to efficiently deliver drugs across the BBB and treat neurological diseases. This review aims to comprehensively summarize the recent advancements in the development of LNPs designed for the controlled delivery and therapeutic modulation of CNS diseases through oral administration. Furthermore, this review addresses the mechanisms by which these LNPs overcome biological barriers and evaluate their clinical implications and therapeutic efficacy in the context of oral drug delivery systems. Specifically, it focuses on LNP formulations that facilitate oral administration, exploring their potential to enhance bioavailability, improve targeting precision, and alleviate or manage the symptoms associated with a range of CNS diseases.

## 1. Introduction

Central nervous system (CNS) diseases, including Alzheimer’s disease (AD), amyotrophic lateral sclerosis (ALS), Parkinson’s disease, multiple sclerosis (MS), and other related disorders, are the leading cause of disability and mortality worldwide [1], and have a profound impact on both individuals and society [2]. Neuroinflammation is considered a feature of numerous CNS diseases, in addition to other pathological alterations [3]. The overproduction of pro-inflammatory cytokines activates downstream signaling pathways, culminating in neuronal apoptosis and neurodegeneration [3,4]. Indeed, a variety of anti-inflammatory drugs have been shown to ameliorate CNS diseases in animal experiments [5,6,7]. The progression time of CNS diseases, especially neurodegenerative diseases, is generally 2 to 9 years. Extended invasive therapy is linked to decreased patient adherence and increased risks of infection. As a result, oral administration is the mainstay of drug delivery in the clinical management of neurological diseases [8]. Although oral administration is considered a simple and safe route, its efficiency in achieving targeted delivery to the brain is relatively low.

Thanks to the rapid development of nanotechnology in recent years, it is possible to use nano-targeted drug delivery systems for the treatment of neurological diseases [9]. Among the diverse types of nanoparticles, lipid nanoparticles (LNPs) have attracted considerable attention for drug delivery to the brain because of their excellent biocompatibility [10,11,12]. LNPs are characterized by a hollow spherical structure and a lipid layer membrane [13,14,15]. LNPs can be specifically modified to overcome various physiological barriers, including the blood–brain barrier, by incorporating targeting ligands or by using surface modifications. These modifications enable the more efficient brain-targeted delivery of therapeutic drugs, ensuring that they reach their intended sites of action in the central nervous system while minimizing off-target effects. The application of LNPs significantly enhances targeted drug delivery and sustained release, improves pharmacokinetic profiles, and reduces systemic adverse effects. Moreover, the combination of nanotechnology with immunotherapy has facilitated the development of innovative targeted therapeutic approaches for CNS diseases. This article reviews the relative progress that has been made in the field of oral LNPs, highlighting their essential role and current challenges in the treatment of neuroinflammation-related CNS diseases, and also pointing out the limitations and offering suggestions for future research.

## 2. Neuroinflammation in CNS Diseases

The etiology of CNS diseases is intricate, with the underlying molecular mechanism yet to be fully elucidated. CNS diseases, especially neurodegenerative diseases, generally have several hallmark features, including disrupted protein homeostasis, dysfunction of synaptic and neuronal networks, cytoskeletal abnormalities, altered energy metabolism, DNA and RNA defects, inflammation, and neuronal cell death [16]. These pathological processes might be induced by endogenous factors, including gene mutations and protein aggregation, as well as by external factors, such as infection, trauma, and drugs. Among these, neuroinflammation has emerged as the central focus in the study of neurological disorders [17,18]. It can clear toxins, inhibit pathogens, and promote local tissue repair to protect the brain parenchyma. Therefore, an appropriate inflammatory response is thought to be neuroprotective. However, a persistent immune response leads to the disorder of CNS neurochemical processes and aggravates neuronal death. Neuroinflammation, characterized by the involvement of neurons and glial cells, is a robust immune response primarily initiated by microglia and astrocytes in response to external injury to the CNS or activation of the autoimmune response. Under certain pathological conditions, peripheral lymphocytes, monocytes, and neutrophils also have the ability to infiltrate the CNS, thereby aggravating neuroinflammation.

Microglia are resident mononuclear phagocytes in the CNS. Microglia-mediated neuroinflammation represents a prominent characteristic of CNS diseases. Under homeostatic conditions, microglia in the CNS mainly stay in a relatively quiescent state and play an ‘immune surveillance’ function [19]. However, neural injury or pathogen infection lead to the presence of damage-associated molecular patterns (DAMPs) or pathogen-associated molecular patterns (PAMPs) in the brain tissue [20], which can be recognized by relevant receptors on the surface of microglial cells, including Toll-like receptors (TLRs), translocation protein 18 kDa (TSPO), myeloid triggering receptor 2 (TREM2), receptor for advanced glycation end-products (RAGE), and CC chemokine 2 receptor (CCR2), etc. [20,21,22,23]. Upon activation, microglia secrete cytokines and chemokines and concurrently recruit other immune cells to clear pathological factors. Activated microglia can be divided into two subtypes: M1 and M2 [24]. The polarization of microglia into the M1 subtype can be induced by stimuli such as lipopolysaccharides (LPSs) or interferon-γ (IFN-γ). M1-type microglia have pro-inflammatory properties and secrete a large number of pro-inflammatory cytokines, including IFN-γ, tumor necrosis factor-alpha (TNF-α), and interleukin (IL)-1β, as well as chemokines such as CCL2, CXCL9, and CXCL10. They also express inducible nitric oxide synthase (iNOS) and produce reactive oxygen species (ROS) [25,26]. These inflammatory mediators and neurotoxic substances are capable of triggering inflammatory response, resulting in tissue damage and neuronal death. This dynamic feedback loop between damaged neurons and uncontrolled inflammation ultimately contributes to progressive neurodegeneration. Conversely, the M2 subtype is induced by IL-4 and IL-13 [27], and is characterized by the production of anti-inflammatory factors such as IL-10, transforming growth factor-β (TGF-β), nerve growth factor (NGF), and brain-derived neurotrophic factor (BDNF), which have neuroprotective effects [28,29,30].

**Figure 1:**
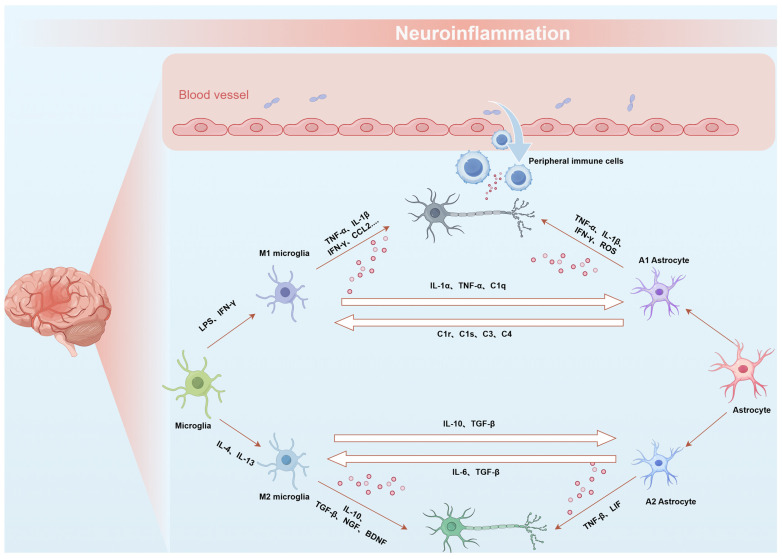
Schematic representation of the interactions between astrocytes and microglia during neuroinflammation. Immune cells, including microglia, astrocytes, and peripheral immune cells, contribute to neuroinflammation through intricate interactions and regulatory mechanisms, forming various feedback loops.

Astrocytes are the most common glial cells in the CNS [31]. Similar to microglia, astrocytes can differentiate into two distinct subtypes: A1 and A2, known as neurotoxic reactive astrocytes and neuroprotective reactive astrocytes, respectively [32]. Furthermore, astrocytes not only directly play an immunological role, but also maintain intricate interactions with neurons and other cellular entities, thereby regulating the pathogenesis of CNS diseases [33]. Liddelow et al. have demonstrated that during the initial inflammatory response, C1q, TNF-α, and IL-1α derived from LPS-activated microglia are both necessary and sufficient for the activation and differentiation of astrocytes into A1 astrocytes [34]. These A1 astrocytes subsequently induce neuronal and oligodendrocyte death, impair the tissue repair capacity, and mediate the late neuroinflammatory response. Therefore, a variety of immune cells, including microglia, astrocytes, oligodendrocytes, and peripheral immune cells, participate in the pathological courses of CNS diseases. These cells engage in complex interactions and mutual regulatory mechanisms, establishing diverse feedback loops [35] (Figure 1).

Currently, no proper therapy has been established to cure CNS diseases and the treatments available are only able to relieve the associated symptoms. However, significant progress has been made in targeting neuroinflammation as a therapeutic strategy against CNS diseases. Thus, further understanding the regulatory mechanism of neuroinflammation is essential to develop CNS-targeted therapies, thereby elevating the precision and efficacy of neurological disorder treatment.

## 3. LNPs for Oral Delivery

Oral administration is widely regarded as the favorable route for the treatment of CNS diseases because of its safety, good patient compliance, and ease of administration. However, many drugs are readily degraded in the gastrointestinal (GI) tract and have difficulty penetrating the GI mucosal barrier, leading to low bioavailability [36]. A critical issue that pharmaceutical researchers always face is determining how to maintain drug stability and enhance drug absorption. Numerous studies have demonstrated that LNPs can efficiently encapsulate a variety of agents, including DNA, RNA, proteins, and small molecules, and protect them from enzymatic degradation in vivo [37,38,39]. LNPs facilitate drug passage through the GI physiological barrier via various transport mechanisms, significantly improving oral drug absorption. Additionally, the surface of LNPs can be modified with specific targeting ligands for precise delivery to disease-specific cells or tissues [40]. Therefore, LNPs hold considerable promise for oral drug administration.

### 3.1. Fundamental Composition and Structural Characteristics of LNPs

LNPs, also known as lipid nanocarriers, are advanced delivery systems [41]. Classical LNPs mainly consist of ionizable lipids, polyethylene glycol (PEG)–lipids, cholesterol, and phospholipids [42] (Table 1). In the LNP delivery system, each lipid excipient serves a distinct function.

**Figure 2:**
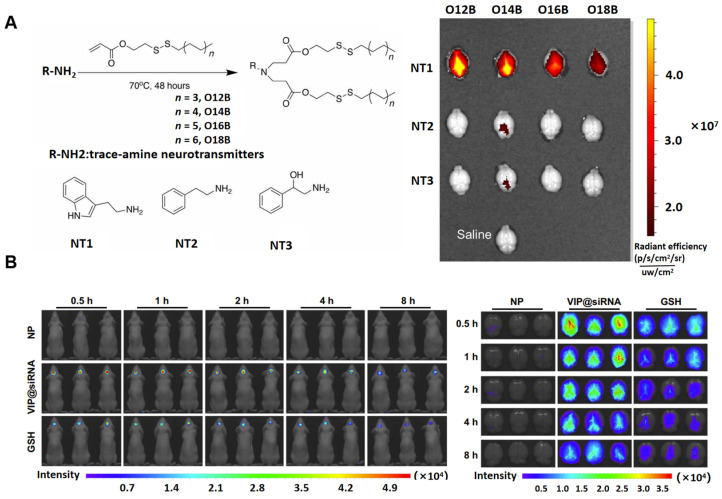
(**A**) Neurotransmitter-derived lipidoids have the ability to facilitate brain delivery. This work was published under the CC-BY-NC license, with copyright held by The Authors, 2020, and was published by The American Association for the Advancement of Science [52]. (**B**) The vincristine-derived ionizable LNP delivery system enables the efficient targeting of drugs to the brain. In vivo imaging of mice was performed at 0.5, 1, 2, 4, and 8 h post-administration to track the LNP distribution. Reproduced under the terms of the CC-BY-NC license, copyright 2024, Springer Nature [53].

Ionizable lipids are critical components in LNP formulations, as their acid dissociation constant (pKa) governs the ionization behavior and surface charge of the LNPs, which in turn influences their stability and toxicity. Traditional cationic lipids with permanent charges often exhibit higher hemolytic activity, increasing the risk of toxic side effects [43,44]. To mitigate these risks, ionizable cationic lipids with pKa values typically ranging from 6.0 to 7.0 have been developed, which significantly reduce toxicity and immunogenicity [45]. These ionizable lipids exhibit the unique ability to preferentially acquire a positive charge under acidic pH conditions, while remaining neutral at physiological pH (7.4). This neutrality at physiological pH minimizes unnecessary electrostatic interactions, ensuring greater stability. Under acidic conditions (pH 4), the ionizable cationic lipids become positively charged, enabling strong interactions with negatively charged payloads [46,47]. Additionally, ionizable cationic lipids facilitate effective intracellular drug release [48]. Under the acidic conditions of endosomes, these lipids acquire a positive charge and interact with negatively charged endosomal lipids, leading to membrane disruption and the release of the drug payload into the cytoplasm [49]. Therefore, incorporating ionizable cationic lipids into LNPs enhances the loading of active pharmaceutical ingredients (APIs) and ensures efficient drug release into the cytoplasm. Exploring the structure–activity relationship (SAR) between ionizable lipids and conjugated targeting groups may provide valuable guidance for the design of LNPs [50,51]. For example, leveraging the permeability of neurotransmitter derivatives, such as serotonin-based compounds, across the blood–brain barrier (BBB) has led to the development of neurotransmitter-derived lipidoid (NT-lipidoid)-based LNPs [52]. These LNPs have demonstrated the ability to successfully deliver small molecules, nucleic acids, and proteins across the BBB, enabling efficient brain-targeted delivery (Figure 2A). Recent research has led to the development of a VIP (vincristine-derived ionizable lipid nanoparticle) delivery system, utilizing the natural compound vincristine. This system ensures both safety and effective brain-targeted drug delivery (Figure 2B). It has demonstrated significant potential in treating brain-related diseases, including AD, brain tumors, and infections, by enhancing the precision of therapeutic delivery to the CNS [53]. The identification of ionizable lipid structures, despite being conceptually guided by rational design, predominantly relies on the random assembly of head groups, linkers, and hydrophobic tails, followed by the selection of optimal lipids based on in vitro and in vivo delivery efficacy. However, limited research has been conducted to elucidate the precise relationship between lipid structure and the delivery performance of LNPs. Consequently, a well-defined theoretical framework is lacking to assist researchers in designing LNPs tailored for targeting specific organs via distinct administration routes for therapeutic applications. To advance this field, both academic institutions and pharmaceutical industries must intensify their efforts to establish a truly rational design approach, deepening the understanding of structure–activity relationships. Such advancements will significantly enhance the development of LNP-based therapeutics.

PEG–lipids, an essential component of LNPs, are composed of PEG conjugated to lipid components. Despite constituting the smallest molar percentage of lipids in LNPs, typically around 1.5 mol%, PEG–lipids exert a substantial impact on the critical properties of LNPs. Specifically, they localize on the surface of lipid particles and maintain the spatial stability of the delivery system. Furthermore, the presence of PEG chains prevents LNP aggregation, serum protein adsorption, and uptake by the mononuclear phagocytic system in vivo, thereby prolonging the circulating half-life of LNPs in the bloodstream [54,55]. Alternatively, extended circulation times lead to a higher number of nanoparticles interacting with the blood–brain barrier. Importantly, the size of LNPs must be carefully controlled during preparation, as it plays a pivotal role in determining their pharmacokinetics, delivery efficiency, and transfection efficacy. Studies have shown that increasing the molar ratio of PEG–lipids results in significantly smaller LNPs, independent of other lipid components [56,57]. These findings underscore the importance of optimizing the PEG–lipid ratio to achieve the desired size and functionality of LNPs. Moreover, beyond their influence on structural properties such as size and stability, variations in the molar ratio and composition of PEG–lipids also markedly affect the biodistribution and cellular interaction of LNPs, which are critical in determining the efficiency of cargo delivery to target cells [58]. PEGylated polymeric nanocarriers were developed by Nance and colleagues for the targeted delivery of the anticancer drug paclitaxel (PTX) to brain tissues. They demonstrated that PEGylation significantly enhanced the brain-targeting ability of the nanocarriers, improving PTX delivery to brain regions compared to uncoated polymeric nanocarriers [59]. Therefore, the careful optimization of these parameters is essential to create a balanced LNP structure that maximizes the therapeutic potential of PEGylated lipids. In conclusion, PEGylated lipids serve a crucial role in enhancing the stability, tissue penetration, and targeting efficiency of LNPs as drug delivery systems. These advancements in PEGylation strategies offer significant potential for developing precise and efficient delivery platforms. Given its widespread application across various drug modalities and delivery systems, PEGylation remains a cornerstone in the refinement of LNP-based therapeutics.

In addition to PEG–lipids, cholesterol, characterized by its rigidity and hydrophobic nature, integrates into the interstitial spaces of lipid membranes, thereby enhancing the stability of LNPs and facilitating their fusion with endosomes [60,61]. Notably, structural modifications of cholesterol can further optimize LNP performance and enable specialized functions. For example, Paunovska et al. demonstrated that esterified cholesterol in LNPs significantly boosted delivery efficiency [62]. Recent research has concentrated on enhancing cholesterol derivatives to optimize LNP performance. These modified cholesterol compounds contribute to the improved stability and prolonged circulation times of LNPs, thereby increasing their delivery efficiency. Choi et al. also achieved a boost in delivery efficiency, though through a distinct approach. Instead of using traditional cholesterol, they employed 3β-L-histidinamide-carbamoyl cholesterol (Hchol), which demonstrated superior delivery and gene expression both in vitro and in vivo. This enhancement was attributed to the pH-sensitive protonation of the imidazole groups in the Hchol formulation [63]. Additionally, the modification of cholesterol’s charge is crucial for targeting. The inclusion of cationic cholesterol in LNPs significantly alters the delivery distribution across various organs. These findings highlight the promising role of cholesterol modifications in improving LNP-based delivery systems, which could pave the way for the development of more advanced therapeutic strategies for various diseases. Phospholipids play a crucial role in the formation of LNPs and facilitate endosomal escape. Commonly used phospholipids such as DSPC and DOPE have been employed in both preclinical studies and clinical applications for years [56]. However, there is a growing need to explore the SAR of phospholipids and optimize their chemical properties to improve their physical and biological functions [50]. To address the limitations of traditional phospholipids, such as their structural rigidity and the difficulty of achieving reaction accessibility, Liu et al. developed multi-tailed ionizable phospholipids (iPhos). The unique structure of iPhos promotes the formation of a cone-shaped structure in the endosomes, which enhances endosomal membrane fusion, facilitates hexagonal phase transformation, and ultimately leads to improved membrane destabilization and efficient cargo release [64]. Furthermore, iPhos-based LNPs have shown potential for targeted delivery, offering significant promise for organ-specific applications [65,66]. In summary, the stereochemical configuration of cholesterol and its derivatives significantly influences the morphology, structural integrity, particle size, and encapsulation efficiency of LNPs, thereby impacting their delivery capabilities. Additionally, phospholipids are essential in LNP design, playing a pivotal role in biodistribution, tissue-specific targeting, and overall therapeutic efficacy. Furthermore, the molar ratio of lipid components is a critical factor in achieving organ-specific delivery and warrants further investigation.

In summary, these studies demonstrate that subtle physicochemical modifications of cholesterol, the ionization state of lipids, or alterations in individual lipid components can significantly impact the structural and biological properties of LNPs. Advanced methodologies, such as large-scale molecular dynamics (MD) simulation and other cutting-edge techniques, hold great potential for guiding the rational design of LNPs to enhance their therapeutic applications.

**Table 1:** Components and targeted delivery strategies of LNPs.

Components of LNPs	Proportion	Representative Components	Types of Targeted Delivery Strategies	Ref.
Ionizable lipids	~50 mol%	SM-102, ALC-0315, 5A2-SC8, D-Lin-MC3-DMA	NT-lipidoid and vincristine-derived ionizable lipid	[52,53]
Cholesterols	~40 mol%	β-sitosterol, 25-hydroxycholesterol, 7α-hydroxycholesterol, 20α-hydrocholesterol	Cholesterol derivatives	[63]
Phospholipids	~10 mol%	DSPC, DOPS, DOPG, 9A1P9, DPOE	Multi-tailed ionizable phospholipids	[64]
PEG–lipids	~1.5 mol%	ALC-0519, DSPE-PEG2000, DMG-PEG2000, polysarcosine-lipid	Covalently bind to the corresponding antibodies or ligands	[59]

### 3.2. Solid Lipid Nanoparticles and Nanostructured Lipid Carriers

**Figure 3:**
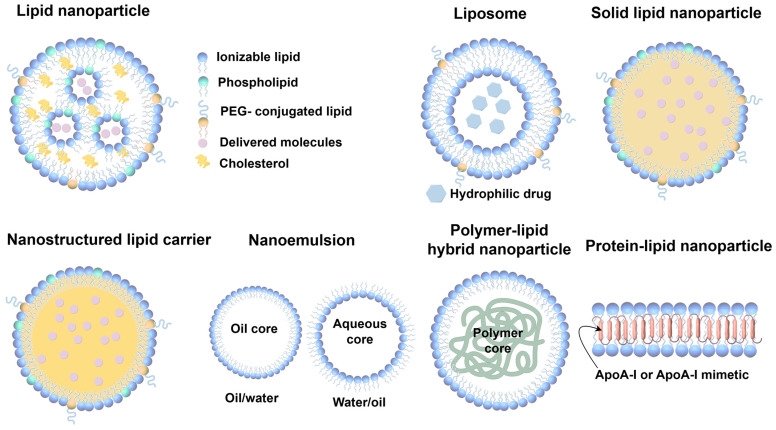
Schematic representation of LNPs. LNPs are composed of ionizable lipids, phospholipids, PEG-conjugated lipids, cholesterol, and delivered molecules; these nanoparticles enable drug encapsulation and efficient delivery. Common structures and components of LNPs: liposomes, solid lipid nanoparticles, nanostructured lipid carriers, nanoemulsions, polymer–lipid hybrid nanoparticles, and protein–lipid nanoparticles.

LNPs are typically classified into several categories, including liposomes, lipid nanoemulsions, solid lipid nanoparticles (SLNs), nanostructured lipid carriers (NLCs), polymer–lipid hybrid nanoparticles (PLNs), and protein–lipid nanoparticles, such as synthetic high-density lipoproteins (sHDLs) [67]. Liposomes, due to their amphiphilic nature, possess both hydrophilic and lipophilic properties, allowing them to efficiently encapsulate and deliver a wide variety of substances with diverse solubility profiles. Multilamellar liposomes, in particular, can incorporate larger amounts of lipophilic molecules into their vesicle membranes, thanks to their increased surface area. Nanoemulsions, on the other hand, are created by dispersing oil droplets in an aqueous phase (*o*/*w*) or water droplets in an oil phase (*w*/*o*), with surfactants stabilizing the system, enabling the encapsulation of either lipophilic or hydrophilic drugs, respectively [68]. Polymer–lipid hybrid nanoparticles (PLNs) are composed of a lipid shell surrounding a polymer core. Protein–lipid nanoparticles, such as synthetic high-density lipoproteins (sHDLs), are structured similarly to endogenous pre-β HDLs [69]. Typically, sHDLs consist of phospholipids and apolipoproteins or their mimetic peptides (Figure 3). In contrast to liposomes, sHDLs usually exhibit a discoidal structure, with the lipid bilayer stabilized by the protein or peptide component. As drug-free nanoparticles, sHDLs can function similarly to endogenous HDLs. Additionally, sHDLs can be utilized as drug delivery systems, as their lipid bilayer structure allows for the encapsulation of lipophilic small molecule drugs [70].

Among these, for the oral delivery of plant-derived bioactive compounds targeting the brain, SLNs and NLCs are more commonly utilized [71]. SLNs feature a solid lipid core composed of biocompatible and biodegradable lipids, such as triglycerides, fatty acids, or waxes, which remain solid at room and body temperature. This solid core provides structural stability and serves as a reservoir for hydrophobic drugs. The particle surface is stabilized by surfactants or phospholipids, enhancing their stability and dispersion [15,72]. SLNs can be converted into solid powder via freeze-drying or spray-drying techniques, thereby facilitating their integration into various dosage forms such as conventional tablets, granules, pills, and capsules [73]. These technological advances also allow for the support of multiple drug delivery methods. Nonetheless, SLNs exhibit certain limitations, including instability during storage [74]. Specifically, the core of solid lipids is susceptible to polycrystalline transition over time, with a substantial proportion of high-energy α crystals with disordered side chains gradually converting into thermodynamically stable β crystals [75]. This transition would lead to reduced drug-loading capacity, increased propensity to aggregate, and abrupt drug release from the SLNs. NLCs represent an advanced class of lipid-based nanodrug delivery systems, developed as an upgrade of SLNs [76]. By partially substituting the solid lipid core with liquid lipids, NLCs diminish the recrystallization tendency of solid lipids, promoting a disordered structural arrangement. This modification helps to increase the drug-loading capacity while mitigating the reduction in nanoparticle stability that is associated with crystal transition during storage. In recent years, newer forms of lipid nanoparticles have emerged, among which smart lipid nanoparticles (smart LNPs) represent an advanced evolution of lipid-based drug delivery systems. Smart LNPs are an advanced form of lipid-based drug delivery systems that are designed to respond to specific external or internal stimuli, such as pH, temperature, enzymes, or ionic concentrations. These stimuli-responsive properties enable smart LNPs to release their encapsulated drugs or therapeutic molecules in a controlled manner at targeted sites, enhancing drug efficacy, reducing side effects, and improving bio-distribution. As a result, smart LNPs provide an advanced, highly efficient drug delivery system with improved loading capacity and better stability during storage, making them a promising solution for delivering drugs with poor solubility [77].

Overall, LNPs enhance oral drug stability, significantly improve bioavailability and targeting efficacy, and possess good development potential. Simultaneously, a comprehensive study of the functions of related LNPs holds substantial significance for the development of LNPs that are more suitable for internal absorption.

## 4. Challenges and Strategies for Oral LNPs in CNS Delivery

The oral absorption of drugs for neurodegenerative diseases is a complex process influenced by multiple factors, with the intestinal epithelial barrier (IEB) and BBB serving as the primary physiological obstacles. This section focuses on the challenges encountered during the oral delivery of LNPs to the brain and discusses corresponding adjustment strategies to enhance their efficacy.

### 4.1. Intestinal Epithelial Barrier for Oral LNPs

Following oral administration, the drugs enter the GI tract, where the complex environment and variable composition of GI fluid significantly affect the stability of the drug carriers. The GI environment, including the mucus barrier, tight junctions, and GI epithelial cells, along with the physicochemical properties of LNPs, such as particle size, charge, surface modification, and lipid matrix, exert varying degrees of influence on the absorption efficacy of LNPs. Therefore, a comprehensive and systematic understanding of the absorption and transport efficiency of LNPs in the GI tract, as well as their mechanisms and influencing factors, are of great theoretical significance for the design and development of novel, efficient, and long-acting oral LNPs.

#### 4.1.1. Gastrointestinal Environment

The pH of gastric juice ranges from 1.0 to 2.5, and that of intestinal juice gradually changes from 6.0 to 7.4 from the duodenum to terminal ileum. These pH fluctuations can induce oxidation, deamidation, or hydrolysis of the drugs, thereby affecting their activity [78,79]. Enzymatic hydrolysis in the GI tract, mediated by enzymes such as pepsin in the stomach, and trypsin, chymotrypsin, elastase, and carboxypeptidase in the small intestine, further affects the structure and function of orally administered drugs [80]. In addition, the GI tract is the main habitat for a variety of microbial flora in the body. These microbial communities and their products may adversely affect LNPs to some extent [81]. To facilitate the translocation of lipid nanocarriers across the intestinal barrier, it is essential to maintain stability within the intestinal environment. SLNs exhibit poor stability under acidic conditions, resulting in significant aggregation in the gastric environment. This limitation hinders their effectiveness as an oral drug delivery system. Polymers such as polylactic–glycolic acid copolymers (PLGA), chitosan, and hydrogels are utilized to enhance resistance to acidic pH conditions [82,83,84,85].

#### 4.1.2. Mucus Barrier

The mucus barrier in the GI tract is a mucus layer that spans the entire tract and is primarily composed of glycoproteins [86]. This layer consists of a tightly adherent component and a loosely adherent component, both of which play critical roles in maintaining GI homeostasis. Beyond glycoproteins, the mucus layer contains various enzymes capable of degrading peptides [87,88]. Its primary function is to protect the underlying epithelium by forming a barrier that prevents pathogens from reaching the epithelial surface and mitigates the effects of digestive enzymes and the acidic GI environment [88]. While the mucus layer plays a protective role for the GI tract, it also acts as an important barrier, impeding the absorption of many drugs [89]. To enhance the absorption of LNPs at the mucosal layer, two strategies have been proposed. The first involves promoting LNP adhesion to the mucus surface, reducing rapid intestinal clearance and extending drug release. The second strategy focuses on enabling LNPs to effectively penetrate the mucus barrier, facilitating faster access to the epithelial surface and subsequent absorption.

Most mucins in intestinal mucus are glycosylated, conferring a negative charge [90]. Enhancing adhesion can be achieved by imparting a positive charge to nanocarriers for electrostatic interactions or through surface modification with specific polymers to optimize nanoparticle design. Several polymers have been identified to improve the mucoadhesive property of LNPs, each with its unique mechanisms and advantages. Chitosan, a biocompatible and biodegradable polycationic polymer, demonstrates exceptional mucoadhesiveness through electrostatic interactions, particularly in acidic environments where its amine groups are protonated [91,92,93]. Thiolated derivatives of chitosan further enhance retention on mucosal surfaces, as shown in studies with insulin and amphotericin B formulations [94,95]. While extensive experience has been gained with chitosan-coated nanoparticles, additional polymers with mucoadhesive potential, such as hyaluronic acid, polyvinyl alcohol, caprolactone, hydroxypropyl methylcellulose, alginate, and pectin, have also been developed to enhance mucus interaction [96,97,98,99]. In addition to surface modification strategies, the size and shape of nanocarriers are critical factors influencing their diffusion through the mucus layer. Generally, nanoparticles that are spherical and within the size range of 200–500 nm are considered more likely to penetrate the mucus barrier [100,101].

Nanocarriers are designed to penetrate the mucus layer effectively, allowing them to navigate through the complex mucus network. This enhanced diffusion not only facilitates their passage through the mucus matrix but also significantly increases their likelihood of reaching the underlying epithelial cells. Hanes’ team pioneered the use of hydrophilic neutral polymer coatings, particularly low molecular weight PEG, to minimize hydrophobic interactions with mucus, establishing PEG as the preferred choice for developing mucodiffusive nanocarriers [102,103,104]. Yuan et al. further demonstrated the efficacy of PEGylation in overcoming mucus barriers, showing that PEGylated SLNs improved the permeability of Caco-2/HT29 co-cultures and enhanced the oral bioavailability of doxorubicin in rats compared to unmodified nanoparticles [104]. Overall, these approaches target the unique properties of the mucus barrier to optimize the therapeutic potential of LNPs in oral delivery.

#### 4.1.3. Intestinal Epithelial Barrier

Beneath the mucosal layer lie the intestinal epithelial cells. These cells form a monolayer comprising tight junctions, adhesive junctions, and desmosomes, which together form the apical junctional complex. This complex serves as a highly selective physiological barrier, preventing the entry of macromolecules and microorganisms into systemic circulation [105].

Glycoprotein (P-gp), an ATP-binding cassette transporter, functions as a critical efflux pump responsible for actively transporting xenobiotics and therapeutic agents out of cells. It is predominantly expressed in cells and tissues such as the intestinal epithelium, blood–brain barrier, liver, and kidneys [106,107], where it serves a protective mechanism. As a major efflux pump, P-gp significantly influences the absorption, distribution, metabolism, and excretion of drugs, thereby affecting the pharmacokinetics and bioavailability of many therapeutic compounds [108]. Its activity represents a crucial consideration in the design of drug delivery systems aimed at overcoming efflux-mediated barriers. To address the limitations of drug efflux mediated by P-gp, various strategies have been employed, including the incorporation of P-gp substrates or inhibitors into nanoparticle formulations. D-α-tocopheryl polyethylene glycol succinate (TPGS) and deoxycholic acid are commonly used intestinal P-gp inhibitors in lipid-based nanocarriers [109,110]. Natural compounds such as myricetin, curcumin, and quercetin have also been shown to have potential as P-gp inhibitors [111]. Nasirizadeh et al. recently reported that SLNs loaded with anticancer drugs exhibited enhanced cytotoxicity against Caco-2 cells when piperine and quercetin were included as P-gp inhibitors. Interestingly, several surfactants frequently used in nanoparticle formulations have been demonstrated to act as low-toxicity P-gp inhibitors [111]. Moreover, excipients such as Cremophor, Tween 80, and PEG inhibit P-gp by altering the membrane’s lipid integrity and fluidity, supporting their utility in enhancing drug delivery efficacy.

After resisting P-gp-mediated efflux, LNPs begin to be absorbed in the intestinal epithelium. If lipid hydrolysis occurs, the resulting degradation products combine with bile salts and phospholipids to form mixed micelles [112]. During this process, drugs encapsulated within the LNPs can transfer to the mixed micelles through passive diffusion, facilitating their absorption by the body. For intact nanoparticles, absorption primarily occurs via the paracellular pathway or transmembrane transport, enabling entry into the lymphatic system and bloodstream. Paracellular transport in the GI tract occurs through aqueous pores between the epithelial cells. However, tight junctions between these cells significantly limit paracellular diffusion [113]. To enhance the paracellular permeability of LNPs, studies have demonstrated that hydrophilic coatings, including surfactants, emulsifiers, and chitosan can reversibly weaken tight junctions, thereby promoting the paracellular transport of LNPs [114,115]. However, only a minor fraction of LNPs traverse the epithelial barrier via the paracellular pathway. LNPs are mainly absorbed by the M cells of Peyer’s patches, a type of specialized intestinal epithelial cell that has a high capacity for transcytosis and the trafficking of particulate substances [116]. M cells phagocytose LNPs and transport them to the basal surface for release. Subsequently, the LNPs enter systemic circulation through the lymphatic vessels. LNPs that enter the lymphatic system can be protected from undergoing hepatic first-pass metabolism, thereby enhancing drug bioavailability. To enhance targeting efficiency, various receptors are expressed on the surface of M cells, including intercellular adhesion molecule (ICAM)-1, l-fucose, β1 integrin, and glycoprotein 2 (GP2). Among the targeting strategies, lectins are widely utilized as ligands due to their reversible binding properties, with wheat germ agglutinin (WGA) and ulex europaeus agglutinin 1 (UEA1) being commonly employed for this purpose [117,118]. Baek et al. demonstrated that the oral administration of paclitaxel encapsulated in hydroxypropyl-β-cyclodextrin-modified SLNs resulted in elevated paclitaxel concentrations in the lymph nodes compared to administration in solution form, highlighting the efficacy of surface modification in promoting lymphatic transport [119]. Another study analyzed the intestinal lymphatic uptake of curcumin from curcumin solution, curcumin-loaded SLNs (C-SLNs), and N-carboxymethyl chitosan (NCC)-coated curcumin-loaded SLNs (NCC-SLNs) in Sprague Dawley rats after oral administration by quantifying the curcumin content recovered from the lymph nodes [120]. The results suggested that C-SLNs and NCC-SLNs manifested significantly higher normalized concentrations of curcumin compared to the curcumin solution. Moreover, NCC-SLNs allowed for further enhancement of the lymphatic uptake of SLNs.

### 4.2. Systemic Circulation

LNPs possess high free energy due to their small size and large surface area, which promotes their interactions with biological macromolecules in biological fluids. Upon entering systemic circulation, NPs acquire a protein corona (PC), a layer of adsorbed proteins that alters their biological identity [121]. Protein adsorption on LNPs depends on both the surface charge and the type of coating used. Studies have shown that high surface charge density increases protein adsorption, while specific coatings, such as Poloxamers, can enhance selective protein adsorption [122] and facilitate the BBB uptake process [123]. Conversely, proteins from the complement system, known as opsonins, mark LNPs for recognition by the mononuclear phagocytic system (MPS), thereby accelerating their clearance and reducing their systemic circulation time [124].

To avoid rapid opsonization and MPS clearance, stealth NPs have been developed using PEG coatings, which delay PC formation and extend NP half-life in circulation. However, PEGylation has limitations, such as anti-PEG antibody production and reduced cellular internalization. Alternative strategies, including stimuli-responsive PEG derivatives, have been explored to address these challenges [125]. Other strategies have also been proposed to reduce or avoid PC formation, such as coating NPs with substances like glutathione or functionalizing their surfaces with polymers to minimize non-specific interactions [126]. Synthetic polymer conjugates, like amphiphilic N-(2-hydroxypropyl) methacrylamide (HPMA) copolymer, can provide NPs with an “invisibility cloak” against serum proteins [127]. Recent advancements suggest that using cell membrane fragments as nanoparticle surface coatings can enhance their circulation time and targeting capability [128]. These hybrid nanomaterials combine the properties of biological cell membranes with those of synthetic nanoparticles [129]. This approach provides immune camouflage, reduces leukocyte uptake, and offers the potential to target non-immune cells like astrocytes and neurons. Membrane-coated nanoparticles and cell membrane-derived nanovesicles are emerging strategies for novel diagnostic and therapeutic applications across various diseases.

### 4.3. Blood–Brain Barrier for Oral LNPs and Strategies to Enhance Transport Efficiency

#### 4.3.1. Blood–Brain Barrier

**Figure 4:**
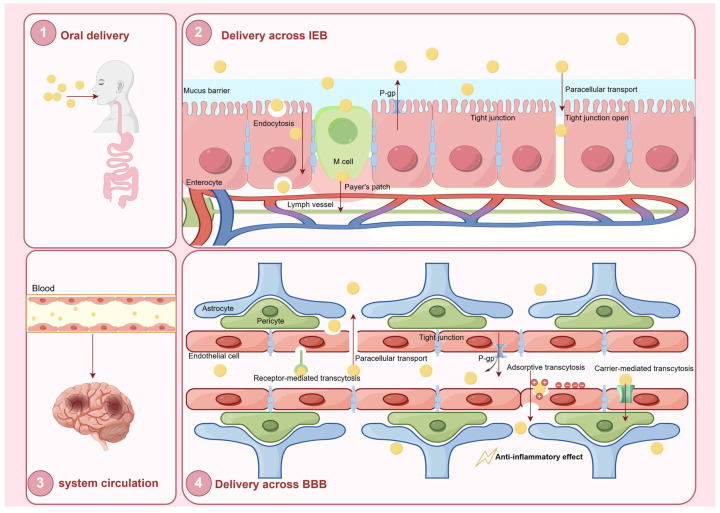
Schematic representation of the oral delivery and systemic circulation of LNPs for CNS drug delivery. (**1**) Oral delivery: LNPs are administered orally and enter the gastrointestinal tract. (**2**) Delivery across the intestinal epithelial barrier: LNPs cross the mucus barrier and enter enterocytes via endocytosis or paracellular transport. (**3**) Systemic circulation: Once absorbed, LNPs enter the blood circulation and are transported toward their target sites. (**4**) Delivery across the blood–brain barrier (BBB): LNPs interact with the endothelial cells of the BBB and traverse the barrier through multiple mechanistic pathways. The primary objective of this process is to facilitate targeted drug delivery to the central nervous system, thereby exerting therapeutic anti-inflammatory effects.

There are two barriers that exist between the circulatory system and the CNS: the BBB and the blood–cerebrospinal fluid barrier (BCSFB). Due to their unique protective characteristics, they play a pivotal role in maintaining brain homeostasis and regulating the transport of ions and molecules [130]. The structure comprises brain endothelial cells, pericytes, astrocytes, and neurons, which establish tight junctions (Figure 4). These cells and their junctional complexes regulate the BBB’s permeability, thereby maintaining the functional integrity of brain and CNS. However, the BBB and BSCFB impede the CNS delivery of more than 90% of small molecule drugs and nearly all biotherapeutic agents [131]. In addition, efflux pumps located within the BBB actively transport drugs back into the bloodstream, thereby preventing these drugs from reaching therapeutically effective concentrations in the brain [132].

There are major challenges in treating CNS diseases by the oral route, primarily due to limited oral absorption, short plasma half-life, and the restrictive nature of the BBB. However, with the development of nanotechnology, the implementation of nanocarrier systems help drugs to cross the BBB and achieve intra-brain delivery.

#### 4.3.2. BBB Transport Mechanisms

LNPs circulate through the vascular network and accumulate at the brain endothelial cells, where they initiate particle-cell binding. Similarly to the intestinal epithelium, the co-delivery of LNPs with P-gp inhibitors can prevent efflux by the brain endothelial cells, thereby enhancing the efficiency of nanoparticle-based drug delivery to the brain. LNPs traverse the BBB through mechanisms similar to those used in crossing the intestinal epithelium, primarily involving paracellular transport and transcellular transport. As mentioned above, SLNs and NLCs are commonly used nanocarrier systems characterized by a low toxicity, high drug-loading capacity, and structural stability [133]. These properties make SLNs and NLCs superior to emulsions, micelles, polymeric nanoparticles, and liposomes for the oral delivery of drugs to the brain.

In terms of paracellular transport, LNPs can be engineered to temporarily disrupt the BBB by employing ligands and molecules. These include cell-penetrating peptides (CPPs), hyperosmotic agents, surfactants (e.g., polysorbate 80), and amino acids (AAs) [134,135]. While this strategy is effective in facilitating NP passage through the BBB, the disruption of tight junctions and adherent junctions (AJs) between the endothelial cells can only be temporary and is typically limited to small NPs (<20 nm), which can exploit this pathway. Moreover, the ligands used to disrupt the BBB are often non-specific and may pose neurotoxic risks when applied over an extended period.

Although small molecules can cross the BBB via passive diffusion, LNPs require transcytosis for entry. Transcytosis is a specific form of transcellular transport in which substances are internalized by cells through endocytosis, transported within vesicles, and subsequently released on the opposite side of the cell. The brain endothelium inherently possesses several transcytosis pathways, including carrier-mediated transcytosis (CMT), adsorptive-mediated transcytosis (AMT), and receptor-mediated transcytosis (RMT), each specialized for the transport of distinct classes of macromolecules [136].

CMT utilizes the brain’s endogenous transporters, which are essential for the regulated entry of molecules across the BBB. Several established carrier systems involved in the transport of various molecules across the BBB include the L-type amino acid transporter (LAT1), glucose transporter (GLUT1), monocarboxylate lactate transporter (MCT1), cationic amino acid transporter (CAT1), choline transporter (ChT), and the sodium-coupled glucose transporter family (SGLTs) [137]. In the design of nanoparticle drug carriers, substrates transported by solute carriers can be utilized as ligands for surface modification to facilitate BBB crossing. For example, Vyas et al. developed phenylalanine-modified SLNs to enhance the brain penetration of efavirenz. In vivo results demonstrated that these phenylalanine-modified nanoparticles accumulated in the brain at levels 3 times higher than unmodified nanoparticles and 7 times higher than the free drug [138]. In addition, PEG functions as a crucial linker in nanoparticle surface modification, promoting the conjugation of ligands or substrates while preserving their spatial orientation. By extending these functional moieties away from the nanoparticle core, PEG enhances their conformational flexibility, thereby improving the efficiency and specificity of their interactions with target transporters [137].

ATM involves the electrostatic interaction between cationic compounds and the negatively charged endothelial cell membrane, leading to the formation of endocytic vesicles [139]. This pathway enables the transcytosis of certain molecules across the BBB using electrostatic interactions. Various drug delivery systems have been developed by utilizing molecules with cationic surfaces, such as chitosan [140]. These systems leverage the positive charge of cationic molecules to enhance interactions with negatively charged cellular membranes, improving drug uptake and delivery efficiency.

Receptor-mediated transcytosis (RMT) facilitates the transport of endogenous macromolecules into the CNS using receptors expressed on the luminal plasma membrane of brain endothelial cells [141]. The key receptors involved in this pathway include the transferrin receptor (TfR), low-density lipoprotein receptor-related proteins 1 and 2 (LRP-1 and LRP-2), insulin receptor, and folate receptor [142]. This mechanism enables the selective entry of essential molecules into the CNS. Relevant ligands can be conjugated onto various nanoparticle carriers to enhance drug delivery, improve BBB penetration, and accelerate transport [143]. However, since TfR itself also undergoes RMT, the drug delivery process may face competitive inhibition, potentially reducing the efficiency of targeted transport.

All the challenges associated with delivering oral LNPs to the brain, along with the corresponding strategies for overcoming them, are summarized in Table 2.

## 5. The Potential of LNPs for Brain Targeting

**Figure 5:**
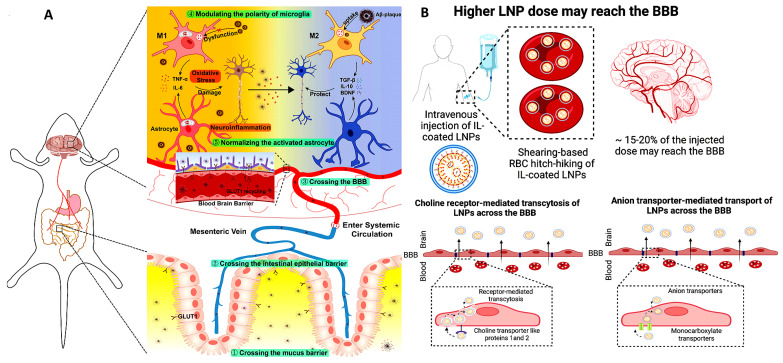
(**A**) Schematic diagram of nanoparticles achieving brain-targeting via oral administration. A smart oral brain-targeting nanoparticle was developed for the multitargeted treatment of Alzheimer’s disease. This nanoparticle was loaded with fingolimod (FTY) and externally functionalized with mannose, incorporating a glucose control strategy to enhance therapeutic efficacy. Reprinted with permission from ref [145], copyright © 2024, American Chemical Society. (**B**) The proposed mechanisms by which IL-coated LNPs are redirected to the BBB following administration. Copyright © 2023, Elsevier B.V. All rights reserved [146].

In addition to the targeting of LNPs through lipid composition modifications, as previously discussed, another strategy involves attaching specific ligands or antibodies to LNPs that bind to cell receptors, enabling targeted delivery to organs that are difficult to reach. AMT relies on nonspecific electrostatic interactions, where polycationic molecules bind to the negatively charged components of the plasma membrane. In contrast to RMT, AMT lacks specificity and may result in non-selective absorption [139]. As a result, non-invasive methods such as oral administration, RMT, and CMT hold great promise. These approaches have the potential to enable the brain-targeted delivery of a wide range of drug formulations. Crossing the BBB through receptor-mediated endocytosis or transcytosis has the potential to enhance the accumulation of brain-targeted LNPs. Receptors such as the TfR, LRP-1, LRP-2, and insulin receptor are present on the luminal side of the BBB [142]. Johnsen et al. developed transferrin receptor-targeted immunoliposomes to facilitate cargo transport across the BBB into the brain parenchyma [143]. Similarly, Kumari et al. investigated lactoferrin-coated nanoparticles for delivering temozolomide, a small molecule drug, across the BBB through receptor-mediated transcytosis [144]. Oral brain-targeting drug delivery remains a significant challenge due to the presence of multiple physiological barriers, with limited studies having addressed this issue. A recent study explored a novel strategy by leveraging the shared characteristics of intestinal epithelial cells and brain endothelial cells, specifically targeting GLUT1, which is highly expressed in both barriers. To achieve co-targeting of the IEB and BBB, α-mannopyranoside was conjugated to the terminal end of PEG. Additionally, a glycemic control strategy was employed to facilitate GLUT1 translocation from the luminal to the abluminal membrane, enhancing the transport of mannose-modified nanoparticles across the BBB and promoting their accumulation in the brain (Figure 5A). This innovative approach successfully enabled oral brain-targeting drug delivery, providing a promising strategy for the non-invasive treatment of neurological disorders [145].

Another approach to brain targeting involves coating LNPs with ionic liquids (ILs), such as choline carboxylates, enabling red blood cell (RBC) hitchhiking. Ionic liquids can potentially alter the biodistribution of LNPs, redirecting them toward the brain. Recently, ILs have emerged as a promising class of biomaterials capable of significantly enhancing the delivery of therapeutics and molecules that typically face barriers through various administration routes, such as buccal [146], intranasal [147], and oral delivery [148]. The process begins by coating LNPs with ILs, which facilitates their adhesion to RBC membranes. Once the IL-coated LNPs are carried by circulating RBCs, they naturally follow the bloodstream’s path toward the brain due to its high perfusion [149]. Upon reaching the brain’s microvasculature, shear forces and endothelial cell interactions cause the IL-coated LNPs to detach from the RBCs. These detached LNPs can then cross the BBB via receptor-mediated transcytosis or other transport mechanisms (Figure 5B). 

## 6. Applications of Orally Delivered Lipid Nanoparticles in Managing CNS Diseases

### 6.1. Alzheimer’s Disease

AD is a progressive neurodegenerative disorder marked by memory impairment, cognitive deterioration, and alterations in behavior. The Aβ peptide activates microglia and astrocytes and initiates an immune response, resulting in the release of pro-inflammatory cytokines, including TNF-α and IL-1β [150]. These cytokines contribute to the recruitment of peripheral immune cells, thereby amplifying the inflammatory cascade and exacerbating the progression of AD [151]. Most AD patients have a prolonged disease trajectory and exhibit a low adherence to treatment regimens. Therefore, oral administration remains the optimal form of home-based medication for them. Resveratrol (RSV) is a natural polyphenol with antioxidant properties and the potential to treat neurodegenerative diseases [152], whose anti-inflammatory effects have been supported by multiple in vivo and in vitro studies. Specifically, RSV prevents IL-2 and IFN-γ production by lymphocytes and inhibits TNF-α and IL-12 secretion by macrophages [153]. However, its poor water solubility, low oral bioavailability (less than 1%), and rapid metabolism limit its therapeutic efficacy. To address these limitations, RSV-encapsulated SLNs (RSV-SLNs) were developed through solvent emulsification and evaporation techniques. RSV-SLNs not only reduce lipid peroxidation levels and increase glutathione levels more effectively, but also mitigate pericapillary congestion and inflammation, and augment its neuroprotective effects in the fundamental processes of AD [154]. Similarly, the natural polyphenol chrysin exhibits a range of biological activities, including anti-oxidative, anti-inflammatory, and neuroprotective effects [155]. Chrysin encapsulated in SLNs (CN-SLNs) showed a higher bioavailability compared to direct oral administration. Notably, oxidative stress and nerve damage induced by Aβ25–35 were significantly attenuated even at reduced oral doses of CN-SLNs. Further GFAP immunohistochemical staining showed that the proliferation of astrocytes in the hippocampus was significantly reduced, while the learning and memory ability were significantly improved in the CN-SLN-treated rats [156]. Curcumin is another plant-derived polyphenol. It has been shown to reduce the levels of pro-inflammatory mediators such as IL-1, IL-6, TNF-α, and iNOS in both animal and clinical studies [157]. These properties confer curcumin with potential preventive and therapeutic effects [158]. However, the oral bioavailability of curcumin is less than 1% due to its poor water solubility and rapid metabolism. At a dose of 50 mg/kg, the learning ability of mice in the SLN group was significantly improved compared to the group receiving curcumin alone. The distribution of curcumin–SLNs was further investigated using fluorescent curcumin nanoparticles and confocal microscopy, and these nanoparticles were found to be present in both plasma and brain tissue. This finding suggests that the oral administration of SLNs facilitates the effective penetration of the intestinal wall and BBB. Notably, the accumulation of curcumin in the brain was observed to increase 30-fold in the SLN group relative to the group receiving oral curcumin alone [159]. Based on the findings of this study, subsequent studies showed that modifications, including non-ionic surfactants (Brij78, TPGS), N-trimethyl chitosan (TMC), PEG, etc., could improve the oral bioavailability of curcumin-loaded SLNs [160]. Berberine, a bioactive alkaloid, shows promise in Alzheimer’s disease (AD) treatment due to its neuroprotective effects, including acetylcholinesterase inhibition, antioxidative activity, and Aβ reduction. However, its poor oral bioavailability (<1%) limits therapeutic efficacy. To address this, berberine-loaded nanostructured lipid carriers (Berb-NLCs) were developed and optimized using a 3² factorial design. The formulation was characterized for physicochemical properties, stability, and drug release, with in vivo pharmacodynamic studies in an Albino Wistar rat AD model confirming its potential for enhanced therapeutic performance [161]. Asiatic acid (AA), a triterpenoid derivative of Centella asiatica (CA), exhibits neuroprotective effects. Liposomes, as nano-vesicular systems for targeted drug delivery, can protect drugs from external degradation and enhance bioavailability by bypassing liver metabolism. However, conventional liposomes are unsuitable for oral delivery due to their rapid degradation in the acidic gastric environment and by gastrointestinal enzymes. To overcome this limitation, liposomes can be modified with polymers, macromolecules, polysaccharides, antibodies, or aptamers to improve brain-targeted delivery and prolong circulation time. This study aimed to develop an ideal oral drug delivery system for treating Alzheimer’s disease (AD) by formulating chitosan-embedded liposomes containing Centella asiatica extract (CLCAE) [162]. Similarly, the researchers examined the effects of liposome-encapsulated thymol (LET) in a rat model of Alzheimer’s disease (AD). The results revealed that LET significantly lowered the levels of pro-inflammatory markers, including IL-1β, IL-6, TNF-α, and COX-2, in both the serum and hippocampus of the rats. These findings suggest that LET may help to mitigate neuroinflammation associated with AD, highlighting its potential as a therapeutic agent.

Currently, in the context of AD, oral LNPs primarily incorporate natural small molecules. These LNPs enhance drug bioavailability and extend the action time of the drugs, thereby potentially improving therapeutic outcomes over conventional medical treatments. In recent studies, researchers have employed short peptides derived from Aβ protein to modify the surfaces of liposomes. This modification facilitates the binding of liposomes to apolipoproteins in the plasma, which then interact with transporters at the BBB, allowing therapeutic delivery to the brain [163]. Researchers have also designed a nanoparticle to penetrate the complex physiological barriers associated with oral administration to the brain, thereby achieving the goal of multitarget therapy for AD. This innovation takes advantage of the absorption characteristics of natural physiological barriers. The nanoparticles utilize PLGA-PEG as a nanocarrier framework, which is selected for its hydrophilic and negatively charged properties to facilitate rapid penetration through the mucus barrier and reduce clearance by the MPS, thereby prolonging the systemic circulation time of the nanoparticles. Furthermore, the nanoparticles are engineered to target GLUT-1 on both the intestinal epithelial cells and brain endothelial cells by grafting α-mannopyranoside to the PEG terminus. This dual targeting strategy improves delivery efficiency to the brain. Upon reaching the target site, the nanoparticles release sphingomyelin, which modulates the activation of the microglia and astrocytes. Microglia shift from a pro-inflammatory M1 phenotype to an anti-inflammatory M2 phenotype, thereby decreasing the release of pro-inflammatory factors from astrocytes while increasing the release of anti-inflammatory and neuroprotective factors. Thus, this approach mitigates neuroinflammation and oxidative stress, ultimately improving the brain microenvironment [145]. These results provide valuable insights into the development of targeted therapy. However, most of the current studies mainly focus on the broad-spectrum effects of these drugs. Further studies are required to explore the potential of oral LNPs to target immune cells during the progression of AD.

### 6.2. Parkinson’s Disease

Parkinson’s disease is a common age-related neurodegenerative disorder affecting the CNS. It is characterized by the progressive degeneration of dopaminergic neurons within the nigrostriatal pathway, leading to a reduction in synaptic dopamine levels, which are crucial for the regulation of a range of motor and non-motor functions. In addition to genetic predispositions and environmental influences, neuroinflammation is increasingly recognized as an important contributor to the pathogenesis of Parkinson’s disease. Apomorphine is a dopamine agonist administered through intermittent subcutaneous injection or continuous infusion to control motor fluctuations in patients with Parkinson’s disease [164]. However, due to the short duration of action, frequent injections are required for apomorphine, often more than 10–15 times per day [165].

In response to this situation, SLNs that encapsulate apomorphine have been developed. Following oral administration, the bioavailability of the drug was significantly increased. The result of drug distribution within the brain indicated that apomorphine could be detected in different brain regions. Compared to aqueous solutions, apomorphine delivered via SLNs showed preferential targeting to the cerebellum, brainstem, and striatum with relatively elevated concentrations, resulting in a marked improvement in the learning ability of mice [166].

### 6.3. Multiple Sclerosis

MS is a neurological disorder that involves the CNS and is characterized by myelin degradation, leading to clinical manifestations such as dyskinesia, paresthesia, and fatigue. The pathogenesis of MS is primarily an autoimmune process, where the body’s immune system mistakenly attacks the myelin sheath, causing inflammation and subsequent neuronal damage. This leads to impaired nerve signal transmission and, over time, results in the neurological symptoms observed in MS patients. At present, there is no definitive cure for MS, and existing drug therapies have some limitations. Methylthioadenosine (MTA), a natural metabolite in the polyamine pathway, has demonstrated potent anti-inflammatory effects both in vitro and in vivo. In an LPS-induced mouse model, MTA administration significantly downregulated pro-inflammatory mediators like TNF-α and NO, and upregulated the anti-inflammatory cytokine IL-10 [167]. Additionally, MTA inhibited mitogen-induced blastogenesis, immunoglobulin synthesis, LPS-triggered TNF-α production in macrophages, and the release of inflammatory cytokines by oligodendrocytes, highlighting its broad anti-inflammatory potential [168]. It has been demonstrated that microencapsulation technology used to prepare MTA-loaded SLNs for the oral delivery of adenosine MTA to the brain results in an approximately 4-fold increase in drug bioavailability compared to an equivalent amount of MTA administered orally. Furthermore, this approach significantly ameliorates the symptoms of MS [169]. Dimethyl fumarate (DMF) is also a commonly used oral drug for the treatment of MS. Recent studies suggest that in neurodegenerative diseases, DMF can reduce lymphocyte counts and reduce serum levels of pro-inflammatory cytokines such as IL-6 and IFN-γ. Additionally, DMF has been shown to enhance neurogenesis and promote BDNF-related neuroprotective effect. These functions highlight the potential of DMF in modulating immune responses and supporting neuronal survival, offering a promising therapeutic strategy for pathological conditions characterized by neuroinflammation and neurodegeneration [170]. In a pioneering study, Kumar et al. integrated retinol acetate and cholecalciferol into the SLNs to enhance DMF delivery to the brain. These formulations significantly increased the oral bioavailability and DMF concentration in the brain and improved the neuroprotective effect of DMF. More importantly, this innovative approach allowed for a once-daily administration of DMF to treat MS [171].

### 6.4. Cerebral Ischemia

Cerebral ischemic disease is a leading cause of brain-related mortality. It is characterized by the excessive production of ROS and reactive nitrogen species, coupled with heightened inflammation, which collectively contribute to significant neuronal damage and destruction [172]. Kaempferol, a major flavonoid in Convolvulus pluricaulis, exhibits a neuroprotective effect against oxidative stress, neuroinflammation, neurotoxicity, neurodegeneration, and cerebral ischemia-induced neuronal damage. Due to its poor water solubility, SLNs have been utilized as an efficient delivery system for kaempferol, prepared using stearic acid and polysorbate 80 via ultrasonication. The kaempferol-loaded SLNs (K-SLNs) demonstrated a controlled release profile with an in vitro drug release rate of 93.24% and significant efficiency in crossing the BBB. In a focal cerebral ischemic rat model, orally administered K-SLNs accumulated in the brain cortex, which suppressed pro-inflammatory molecules such as NF-κB and p-STAT3, and reduced neurological deficits, infarct volume, and ROS levels [173]. Hydroxysafflor yellow A (HSYA), another bioactive flavonoid, regulates macrophage function and inhibits the production of pro-inflammatory cytokines IL-1β and IL-6 [174]. Despite its high water solubility, HSYA has poor intestinal membrane permeability, leading to low oral bioavailability. To address this, HSYA-SLNs with a w/o/w structure were prepared using a warm microemulsion technique. This formulation significantly enhanced the oral absorption of HSYA and effectively reduced brain infarct size in the rat model, demonstrating its potential for improving therapeutic outcomes in neurological disorders [175].

### 6.5. Mental Disorders

Mental disorders, also known as psychiatric disorders, encompass a wide range of conditions affecting mood, behavior, and thinking. The pathogenesis of mental disorders is complex, involving a combination of genetic, biological, environmental, and psychological factors. Recent studies suggest that inflammation and immune system dysfunction may play a role in the pathogenesis of various mental disorders. Increased levels of inflammatory markers have been observed in patients with these conditions [176]. Oral pharmacotherapies for depression, schizophrenia, and anxiety often encounter challenges, including limited water solubility, extensive first-pass metabolism, and a short action duration. Therefore, patients with mental disorders often require multiple daily doses, which may lead to reduced adherence to treatment regimens. Thus, the application of nanomedicine in the treatment of mental disorders represents a promising strategy to improve therapeutic outcomes. Thymoquinone (TQ, 2-isopropyl-5-methyl-1, 2-benzoquinone) is the main active compound in the volatile oil of black seed and has been found to have significant anti-oxidative, anti-inflammatory, and immunomodulatory activities [177]. Rats were treated with TQ solid lipid nanoparticles (TQ-SLNs, 20 mg/kg, p.o) and TQ suspension (20 mg/kg, p.o), respectively. Compared with TQ suspension, SLNs increased the intestinal permeability of TQ by about 250% [178], which significantly reduced the elevation of IL-6 and TNF-α levels in the hippocampus, and reversed the hippocampal KYN/TRP and 5HT/TRP ratios in stressed rats [179]. Subsequent studies further showed that the oral administration of LNP-encapsulated drugs could significantly improve their bioavailability. For example, amoxapine (AMX), when used as oral AMX-SLNs, resulted in a 5.8-fold increase in the drug concentration in the brain compared with a formulation using non-lipid nanoparticles [180]. Perphenazine-loaded SLNs (PPZ-SLNs) had a 16-fold increase in the drug concentration in the brain [181]. Additionally, the oral bioavailability of drugs such as venlafaxine, sulpiride, and quetiapine has been demonstrated to be significantly improved following their incorporation into SLNs. Cannabidiol (CBD), due to its high lipophilicity and low aqueous solubility, faces challenges in oral bioavailability, leading to poor intestinal absorption. To address these limitations, a nanoemulsion formulation of CBD was developed for the potential therapeutic use in treating autism spectrum disorder. The results showed that CBD nanoemulsion treatment successfully reversed autism-like behaviors, possibly by protecting against hippocampal neuronal damage. This approach suggests a promising avenue for improving CBD’s effectiveness in ASD treatment [182]. Polymer–lipid hybrid nanoparticles (PLNs), particularly those with a chitosan-coated lipid nanoparticle structure, represent an innovative approach in drug delivery systems. These nanoparticles combine the beneficial properties of lipids with the advantages of chitosan as a polymer, creating a unique formulation. The study focused on developing PLNs with a chitosan coating to serve as an efficient oral delivery system for carbamazepine, aiming to improve the treatment of epilepsy [183]. Table 3 illustrates the use of LNPs in oral delivery for the treatment of CNS-related disorders.

## 7. Limitations of LNP Delivery

In recent years, LNPs have garnered significant attention from researchers due to their potential as drug delivery systems. Thanks to their lipophilicity, these nanoparticles demonstrate the ability to overcome challenging physiological barriers, such as the blood–brain barrier, even without surface modification. However, these lipids can also lead to some side effects. Ionizable lipids are key components of LNPs, but they carry potential toxicity. These lipids can activate Toll-like receptors (TLRs), particularly TLR4, leading to the production of pro-inflammatory cytokines [184]. This immunostimulatory effect has been observed with ionizable lipids such as DLin-MC3-DMA and C12-200. Metabolites derived from ionizable lipids, such as fatty acids, can induce toxicity by activating peroxisome proliferator-activated receptors (PPARs). The activation of these pathways may result in inflammation and liver toxicity [185]. Another potential source of toxicity is PEGylated lipids. The long-term safety of PEGylated lipids is a concern due to their ability to alter the pharmacokinetics and biodistribution of LNPs. Repeated administration of PEGylated LNPs can trigger immune responses, leading to the production of anti-PEG antibodies. These antibodies can accelerate the clearance of subsequent doses of PEGylated LNPs from the bloodstream, reducing their therapeutic efficacy and increasing the risk of adverse reactions due to the rapid and unexpected distribution of the nanoparticles. Furthermore, LNPs are recognized by the body’s immune system as foreign substances, which triggers the innate immune response and subsequently impacts adaptive immunity [186]. The rapid drug release observed with some oral LNPs may raise toxicity concerns, while excessively slow drug release can lead to suboptimal therapeutic effects. Therefore, the development of novel LNP formulations with an optimized release profile is crucial. This depends on the selection of appropriate cargo drugs, as different drugs exhibit distinct physicochemical properties and interactions with nanoparticles. Prolonged drug circulation is a common characteristic of oral LNP delivery; however, this extended circulation can result in slower tissue accumulation, including in the targeted tissues. Consequently, further optimization of LNP formulations is essential for improving their efficacy. Meanwhile, there are currently no clinical trials specifically assessing oral LNPs for CNS disease. This gap in the research highlights an area that warrants further investigation, and we hope that future studies will focus on addressing this issue.

The lack of established regulatory guidelines from the FDA or other regulatory bodies for products containing nanomaterials presents a significant challenge in the approval of nanomedicines for clinical use. Currently, the evaluation of nanomaterials primarily emphasizes toxicity concerns and the potential commercial benefits of nanoparticles, which can delay both the approval process and subsequent market entry. In addition, research on LNPs has largely concentrated on enhancing target specificity and efficient deposition. Multifunctional LNPs, which combine therapeutic efficacy with target specificity, have also been developed. Concurrently, research is ongoing to explore the potential of conjugating LNPs with other drugs and carrier molecules to further enhance therapeutic outcomes. To validate the effectiveness of LNPs, it is essential to develop and assess their safety in various disease conditions, as well as evaluate novel combination strategies through clinical studies.

## 8. Conclusions and Perspective

The delivery of drugs for brain diseases remains a significant challenge due to the protective BBB, which restricts drug penetration into the CNS. Current drug delivery strategies can be broadly categorized into invasive and non-invasive approaches. Invasive methods, such as intravenous, intrathecal, and brain parenchymal injections, face limitations like venous thrombosis, neurotoxicity, and unsuitability for long-term treatment. Among the non-invasive methods, intranasal administration bypasses the BBB and avoids first-pass metabolism, but its clinical utility is hindered by low patient compliance and inconsistent bioavailability. Oral administration, the most common non-invasive route, has gained traction with advances in nanotechnology, particularly through the use of LNPs. LNPs enhance drug bioavailability, offering a flexible platform for targeted drug delivery. Incorporating machine learning into LNP design could revolutionize high-throughput nanoparticle synthesis by optimizing experimental parameters and refining drug delivery systems. Additionally, the integration of single-cell RNA sequencing and spatial transcriptomic techniques can provide detailed molecular maps of brain cell types, guiding the precise targeting of LNP-delivered therapeutics. The intricate communication network between neurons and glial cells highlights the potential for LNPs to impact multiple CNS cell types, yielding broad therapeutic effects. Future studies should investigate the biodistribution of LNPs and their payloads across different cell types under various pathological conditions to tailor treatments effectively.

## Figures and Tables

**Table 2:** Challenges and strategies in oral LNP delivery.

Challenges	Strategies for LNPs	Refs.
Gastrointestinal environment	PLGA, chitosan, and hydrogels (pH conditions)	[82,83,84,85]
Mucus barrier	Promote LNP adhesion: Chitosan, hyaluronic acid, etc. Penetrate the mucus barrier: PEGylation	[94,95,96,97,98,99,100,101,102]
Intestinal epithelial barrier	P-gp efflux pump: TPGS, deoxycholic acid, Tween 80, PEGylation, etc. Paracellular diffusion: surfactants, emulsifiers, and chitosan M cell-mediated phagocytosis: N-carboxymethyl chitosan, and hydroxypropyl-β-cyclodextrin Carriers as ligands for surface modification: lectins, WGA, and UEA1	[109,110,111,112,113,114,115,116,117,118,119]
Systemic circulation	PEGylation, glutathione, HPMA copolymer, and cell membrane fragments as surface coatings	[125,126,127,128,129]
Paracellular transport	CPPs, hyperosmotic agents, surfactants, amino acids	[134,135]
Carrier-mediated transcytosis	Carriers as ligands for surface modification: LAT1, GLUT1, MCT1, CAT1, ChT, and SGLT	[137]
Adsorptive-mediated transcytosis	Chitosan	[140]
Receptor-mediated transcytosis	Key receptors: TfR, LRP-1, LRP-2, insulin receptor, and folate receptor	[142]

**Table 3:** Oral LNP formulations encapsulating drugs for CNS disease treatment: composition and key properties.

Active Ingredient	Indication	Type of LNPs	Surfactants	Lipids	Physicochemical Properties	Ref.
Resveratrol	Alzheimer’s disease	SLN	Tween 80 and soy lecithin	Glycerol monostearate	Size 104.5 ± 12.3 nm, EE% 72.9 ± 5.31%, zeta −3.1 ± 0.15 mV	[152]
Chrysin	Alzheimer’s disease	SLN	Lecithin and sodium taurocholate	Stearic acid	Size 240.0 ± 4.79 nm, EE% 86.29 ± 3.42%, zeta −40.4 ± 2.54 mV	[156]
Curcumin	Improvement in oral bioavailability	SLN	TPGS (P-gp inhibitor) and Brij78	Glycerol monostearate	Size 135.3 ± 1.5 nm, EE% 91.09 ± 1.23%, zeta −24.7 ± 2.1 mV	[160]
Berberine	Alzheimer’s disease	NLC	Tween 20 and solutol HS 15	Solid lipid: stearic acid; liquid lipid: isopropyl myristate	EE% 88%, zeta −36.86 mV	[161]
Asiatic acid	Alzheimer’s disease	Liposome	Chitosan	Phospholipon 90 G	Size 224.4 nm, EE% 51.3 ± 0.03%, zeta −22.3 mV	[162]
Apomorphine	Parkinson’s disease	SLN	Pluronic F68	Glycerol monostearate and polyethylene glycol monostearate	Size 154.97 ± 2.83 nm, EE% 90.38 ± 0.04, zeta −5.63 ± 0.21	[166]
Methylthioadenosine	Multiple sclerosis	SLN	Tween 80	Stearic acid	Size 89.79 ± 4.67 nm, EE% 93.74 ± 5.09%, zeta −8.43 ± 0.63 mV	[169]
Dimethyl fumarate	Multiple sclerosis	SLN	Tween 80 and PL 90 G	Stearic acid and vitamins (cholecalciferol/retinol acetate)	Size 118.8/198.7 nm, EE% 83.99 ± 3.36%/88.22 ± 3.97%, zeta −1.96/−0.309 mV	[171]
Kaempferol	Cerebral ischemia	SLN	Tween 80	Stearic acid	Size 451.2 nm, EE% 84.92%, zeta −15.0 mV	[173]
Hydroxysafflor yellow A	Cerebral ischemia	SLN	Tween 80	Glycerol monostearate	Size 214 ± 13 nm, EE% 54.94 ± 2.36%, zeta −12.4 ± 1.2 mV	[175]
Thymoquinone	Mental disorders	SLN	Tween 80	Glyceryl monostearate and poloxamer 188	-	[179]
Amoxapine	Mental disorders	SLN	Tween 80	Glycerol monostearate	Size 151.5 ± 7.02 nm, EE% 85.8 ± 3.42%, zeta −24 ± 3.05	[180]
Perphenazine	Mental disorders	SLN	Soy lecithin	Glycerol monostearate	Size 110 ± 5 nm, zeta −26.7 ± 1.8 mV	[181]
Cannabidiol	Autism spectrum disorder	Nanoemulsion	Tween 80	Span 80	Size 107.6 ± 1.1 nm, zeta −30.2 ± 1.3 mv	[182]
Carbamazepine	Epilepsy	Polymer–lipid hybrid nanoparticles	Tween 80 and chitosan	Glyceryl tripalmitate		[183]
